# Behavioral Disinhibition Can Foster Intentions to Healthy Lifestyle Change by Overcoming Commitment to Past Behavior

**DOI:** 10.1371/journal.pone.0142489

**Published:** 2015-11-11

**Authors:** Bob M. Fennis, Tor W. Andreassen, Line Lervik-Olsen

**Affiliations:** 1 University of Groningen, Groningen, The Netherlands; 2 NHH, Bergen, Norway; 3 BI, Norwegian Business School & Center for Service Innovation (CSI), Oslo, Norway; Saarland University, GERMANY

## Abstract

To curb the trend towards obesity and unhealthy living, people may need to change their entire lifestyle to a healthier alternative, something that is frequently perceived to be problematic. The present research, using a large, representative community sample, hypothesized and found that a key factor responsible for why people do not intend to change lifestyles is a sense of commitment to past behavior. However we also found that the contribution of commitment was attenuated for individuals with a stronger tendency for behavioral disinhibition thus underscoring the “bright side” of this individual difference characteristic that traditionally has been mainly associated with impulsive and indulging behavior. Overall, the present findings add to our understanding of factors inhibiting and promoting healthy behavior change.

## Introduction

According to the World Health Organization, more than two billion people will be overweight by next year and as many as 700 million of them obese [1, 2]. To curb that trend it might not be enough for people to simply change one or two harmful behaviors, given the multifaceted nature of the problem and its causes, but it may require changing entire lifestyles from sedentary and hedonistic to sustainable and healthy [3]. This, however, is a serious problem for many. Indeed, a large number of studies suggest that changing multiple behaviors in parallel, such as giving up smoking, changing one’s diet, and engaging in physical activity, poses a tremendous challenge for people [4, 5, 6], see also [7] for a recent meta-analysis.

Why is lifestyle change considered to be so problematic? Among the various reasons, one of the more prominent roles is taken by a key constituent of lifestyles–habitual behavior–which makes any behavior change to a positive, healthy alternative a challenge [4, 8, 9, 10]. Indeed, if an engrained behavior pattern prompts one to eat unhealthy snacks and lead a sedentary life, it becomes very difficult to leave the cookie alone and go for the apple or to forego the car and take the bicycle to work [11,12]. With that in mind, changing an entire existing lifestyle into a new, more sustainable, healthy one might seem like an insurmountable hurdle.

In keeping with the gist of previous research, the present work will highlight an as yet unexamined psychological corollary of past habitual behavior that profoundly obstructs the likelihood of future, positive lifestyle change, but will also demonstrate that some people might intrinsically “have what it takes” to overcome this impediment.

### Commitment to past behavior

What is it about habits that make it such an obstacle to lifestyle change? Research has emphasized two essential features that characterize habitual behavior: its *repetitive nature* and the ensuing *automaticity* of habit-induced behavior [9, 13, 14, 15]. That is, research has highlighted that habits can be conceived of as learned mental representations that link specific situational cues to specific behaviors, whose performance becomes automatic over time when triggered by the cue. As a result, simply encountering the situational cue suffices to trigger the associated behavior, and when the situation is encountered frequently, the ensuing behavior is displayed frequently, thus manifesting itself as repetitive, habitual behavior. Hence, when one has come to associate watching a movie at the cinema with eating a bucket of popcorn, then eating a bucket of popcorn will become the ‘default’, automatic, behavioral response displayed each time when one is at the cinema [16].

Interestingly, while the bulk of previous studies has focused on the automatic, effortless and efficient operation of habits in shaping behavior, less attention has been paid to the motivational consequences of past habitual behavior, with one notable exception [14]. This research [14] highlights that an additional key feature of habitual behavior is that it forms part of someone’s personal identity. That is, it is one of the components that people use to define who and what they are in life. As a critical component of one’s identity, people may thus experience a strong sense of commitment to past behavior. Indeed, habitual behavior, and by implication an entire lifestyle of which it forms the central core, may significantly contribute to a sense of uniformity, regularity and coherence [17] and so may well become a strongly valued and meaningful beacon in one’s life.

Commitment to past behavior thus forms a key psychological corollary of (strong) habits and constitutes a “pull of the past” of one’s lifestyle [16] with potentially profound motivational consequences for intentions to change future behavior. That is, research on the influence of commitment on future choice and decision making (regardless of the object of this commitment) has underscored that commitment operates such that it tends to perpetuate past judgments, choices and behavior–if one has displayed behavior A previously, one tends to display behavior A in the future, ultimately paving the way to honoring sunk cost and escalation of commitment [17, 18, 19, 20]. Similarly, we propose that a sense of commitment to one’s current lifestyle will predict a tendency to perpetuate the constituent habitual behaviors and thus will negatively contribute to intentions to change future behavior. In addition, this sense of commitment follows from past habitual behavior forming part of one’s identity. This yields two additional reasons to expect a constraining role of the construct. Because people generally tend to strive to maintain a consistent personal identity [17, 21], and because changing past behavior may directly threaten the integrity of the self, we may expect that commitment to past behavior may become a force significantly inhibiting intentions to future behavior change. Interestingly, this issue has not yet received systematic research attention.

In sum, the obstructing power of habitual past behavior may surface via its motivational consequences, i.e., a sense of commitment to one’s tried and trusted lifestyle. We aim to show that this commitment is an important predictor of the intention to change lifestyle over and beyond established predictors of intentions to change behavior, notably those derived from the Theory of Planned Behavior (TPB; [22, 23, 24])–attitudes, subjective norms, and perceived behavioral control.

### Commitment and behavioral disinhibition

Interestingly, focusing on commitment to past habitual behavior points to new and as yet unexamined factors that may overcome its inhibiting influence on the intention to change lifestyle. Previous research has focused on contextual, situational influences on the formation and perpetuation of (sometimes risky or unhealthy) habitual behavior and, consequently, has reverted to situational factors as well when it comes to exploring the potential for behavior change, such as inducing implementation intentions [25, 26], the use of simple decision heuristics [27] or changing the “choice architecture” through the use of so-called “nudges” [28]. Complementing that reservoir, the present perspective highlights an intrapersonal, rather than situational, force underlying habitual behavior, and so we also zoom-in on an intrapersonal avenue that might counter the influence of past habits.

More in particular, to overcome the paralyzing effects of commitment to past behavior patterns on lifestyle change we focus on a specific, but perhaps unlikely candidate–individual differences in *behavioral disinhibition*, or the chronic tendency to “let go”. More specifically, we connect with an established framework on behavioral inhibition that comprises two systems of self regulatory behavior–the behavioral inhibition system (BIS) and behavioral activation system (BAS) [29]. Behavioral inhibition entails suppressing behavior that might have aversive consequences, while behavioral activation captures appetitive, approach oriented, goal-directed behavior toward potentially rewarding stimuli (see also [30, 31, 32]). While strong BIS is related to reflective, and deliberate judgment and choice and may extend to anxiety-related disorders [30], weak BIS (also labeled strong *dis*inhibition, as we also do in the present work) is associated with lower levels of impulse control and hence no or only a weak tendency to withhold a prepotent behavioral response [33].

Behavioral disinhibition might be considered an unlikely candidate, as it is associated with increased impulsivity and risk behaviors such as binge eating and drinking, substance abuse and gambling [34, 35, 36, 37]. Yet, in the present context, disinhibition may actually have a “bright side”–if commitment to past behavior inhibits positive behavior change to a healthier lifestyle, then behavioral disinhibition may do the opposite and so may overcome the inhibiting effect of commitment on intentions to change lifestyle.

However, an alternative–opposite–case can also be made, i.e., that disinhibition would actually *boost* rather than *attenuate* the inhibiting contribution of commitment based on the logic that commitment is part of habitual behaviors and habits form a class of prepotent response tendencies whose expression might be more likely under conditions of high disinhibition. Yet, the former possibility might be more plausible than the latter. More specifically, research suggests that a key process responsible for why disinhibition fosters impulsive behaviors is that it ‘frees a person from situational constraints’ [32]. A direct implication from this conceptualization is that situationally constrained behaviors have a lower likelihood to be expressed under these conditions. As discussed above, habits can be conceived of as situationally constrained behaviors, where specific situational cues automatically trigger associated behaviors. Hence, when disinhibition indeed reduces ‘the power of the situation’, then by extension it should also reduce (rather than boost) the contribution of habits’ motivational corollary, commitment to intentions to change lifestyle. The present research will explore both options on the role of disinhibition.

Given that behavioral (dis)inhibition is part of a dual self-regulatory system, the critical reader might wonder whether behavioral activation might have a similar predictive value as behavioral disinhibition. There are reasons to think it might not. More specifically, BAS, with its three constituent dimensions (drive, fun seeking and reward responsiveness) is distinct from BIS in that its approach orientation by necessity requires a target to move towards (cf. [29]). BIS, however, prompts inhibition, inertia, not moving, which does not necessitate a target. By implication, BAS is target-constrained and domain-specific, while BIS (and by extension also its antipode, disinhibition) is target-free and domain-general. These features might give behavioral disinhibition a “competitive edge” compared to BAS in the present context where we focus on the intention to change a *general* lifestyle rather than a highly targeted, specific behavior. Hence, we expect a tendency for disinhibition, rather than any of the three subtypes of behavioral activation, to be particularly able to overcome the inhibitory role of commitment to past behavior in fostering intentions to lifestyle change. Stated more formally, we expect the negative effect of commitment to past behavior on intentions to healthy lifestyle change to be moderated by behavioral disinhibition, such that its predictive value is largely attenuated for individuals with a higher inclination for behavioral disinhibition.

In sum the present work has three key objectives. First, to extend previous work on the role of habits in predicting lifestyle change by focusing on commitment to one’s current lifestyle as a key hindrance to intentions to change lifestyles. Second, to underscore “the bright side” of behavioral disinhibition by showing that this stable individual difference characteristic (rather than any of the BAS dimensions) may be able to overcome the inhibition to change induced by this commitment. Finally, we will extend previous research which has typically relied on undergraduate students, convenience samples, or highly motivated, health-oriented participants, by examining our notions using a large, representative community sample.

## Method

### Participants

To make sure that sample size was sufficient to warrant satisfactory statistical power (cf. [38]), a total of 1000 people (481 women) with a mean age of 48.02 years (*SD* = 16.20) participated voluntarily in the present study which was part of a larger project on values and lifestyles. Data collection was performed by Norstat, a large market research firm based in Norway. The sample was drawn from a large survey panel maintained by the company, which is representative of the Norwegian population.

### Procedure

Participants were approached online and asked to complete a survey on “changing lifestyle to a more sustainable lifestyle”, defined to participants as “all the activities you do to promote a healthy living and to promote your individual well-being. This includes a way of life which involves having the right amount of food, drinking, and exercises so that you as a person, are in a state of physical, social and mental well-being, whilst having the ability to meet the demands of the environment without undue fatigue” (see S1 Text: Full questionnaire and instructions).

### Questionnaire

#### Intentions to lifestyle change

In line with previous research [39] the intention to change to a new lifestyle was measured using the following three 7 point Likert statements (1 = strongly disagree, 7 = strongly agree): “I would like to adopt a new lifestyle today, if possible”, “I will try to adopt one new element of a new lifestyle as soon as I can” and “I am likely to be one of the first of my friends to adopt a new lifestyle”. Reliability of the measure was highly satisfactory (*α* = .84). An index of the intention to change lifestyle was created by averaging scores on the items with higher scores indicating a higher intention.

#### Commitment

We measured commitment to people’s habitual behavior using two 7-point Likert statements rating the extent to which participants valued their current lifestyle more than any new lifestyle and the extent to which their current lifestyle meant a lot to them. An index of commitment was created by averaging the scores on the items with higher scores indicating a higher commitment (*α* = .88).

#### TPB predictors

The three established predictors of intention to change, derived from the Theory of Planned Behavior (attitude, subjective norms, and perceived behavioral control, [22, 23]) were included to assess whether commitment to past behavior can be shown to have any incremental predictive value above and beyond these (cf. [24]). *Attitude* was measured using six 7-point semantic differential scales assessing the extent to with participants rated the lifestyle change as good vs. bad, favorable vs. unfavorable, pleasant vs. unpleasant, harmful vs. beneficial, punishing vs. rewarding and foolish vs. wise (cf. [40]; *α* = .88). *Subjective norm* was measured using the following three 7-point statements: “With respect to adopting a new lifestyle, I would like to do what my closest friend(s) think(s) I ought to do” (1 = not at all, 7 = very much), “Regarding adopting a new lifestyle, I want to do what my friend(s) think(s) I should do” (1 = unlikely 7 = likely) and “How much do you want to do what your closest friend(s) think(s) you should do?” (1 = not at all, 7 = very strongly, *α* = .89). *Perceived behavioral control* was measured using eight 7 point Likert scales (1 = fully disagree, 7 = fully agree, *α* = .92) rating the extent of a sense of self-efficacy (cf. [41]). Sample items include “When facing difficult tasks. I am certain that I will accomplish them” and “In general, I think that I can obtain outcomes that are important to me”. Please note that these items tap into more general, rather than specific perceptions of behavioral control. We included this measure because the focus in the present research is on an *entire* lifestyle rather than on one specific target behavior (e.g., taking the bicycle to work instead of the car). With that in mind, and in line with TPB’s ‘correspondence principle’ [42], one would expect a *general* indicator to be a particularly suitable candidate to predict a similarly *general* outcome and so it makes sense to measure perceived behavioral control on a general, rather than specific level. For all three constructs indices were created by averaging the scores on the items with higher scores indicating higher levels of each of the constructs.

#### Behavioral disinhibition

Participants completed the full BIS/BAS scales developed by Carver and White [29]. Behavioral disinhibition was measured by creating an averaged index of the seven items of the BIS/BAS scales used to measure behavioral inhibition (*α* = .77). The BIS/BAS scales use 4-point scales with 1 = very true for me, 4 = very false for me, but for the present purposes we reversed the scores on the pertinent BIS items so that higher scores indicate higher disinhibition. Sample items from the BIS include “Even if something bad is about to happen to me, I rarely experience fear or nervousness” and “I have very few fears compared to my friends”. To examine whether behavioral activation might play a similar role as that postulated for behavioral disinhibition, each of the three BAS dimensions (BAS drive, fun-seeking and reward responsiveness) was created by averaging the pertinent items with higher scores indicating a higher level of each of the constructs (*α* = .73, .68 and .88, respectively).

## Results

### Descriptive statistics and correlations

A total of *N = *400 participants (40% of the sample) were educated at the high school level or lower, *N = *363 (36.3%) completed 3-year college, while *N =* 237 (23.7%) completed a postgraduate/Master program. In addition, 31.3% of our sample earned an annual income of up to $45.825 (350.000 NOK), 43.4% between $45.825 and $72.011 (550.000 NOK), 15.6% between $72.011 and $98.197 (750.000 NOK), 4.7% between $98.197 and $124,383 (950.000 NOK), and 5% over $124,383. Of our total sample, 21.2% was unmarried, 66,1% was married/cohabitant, 9.6% divorced, and 3.1% widow/widower.

Descriptive statistics and correlations of the variables under study are presented in Table 1. On average, participants showed a modest intention to change to a new lifestyle (*M =* 3.25, *SD =* 1.40) and a fairly high extent of perceived behavioral control (*M =* 5.08, *SD =* .93).The extent of behavioral inhibition on average was only slightly above the midpoint of the scale (*M* = 2.21, *SD =* .50). Of the TPB control variables, attitude and subjective norm were positively correlated with intention to change lifestyle, thus replicating previous research, while perceived behavioral control appears unrelated. Interestingly, in line with predictions, commitment to past behavior was negatively correlated with intention to change lifestyle. Finally, illustrating and corroborating the negative role that disinhibition has been found to play in health-related behavior in previous research, this construct was modestly, but negatively, correlated with intention to adopt a healthy lifestyle, and the three TPB predictors and positively with perceived behavioral control and commitment to past behavior.

**Table 1:** Means, standard deviations and correlations of the main variables under study.

	*M*	*SD*	(1)	(2)	(3)	(4)	(5)	(6)
Lifestyle change (1)	3.25	1.40	-					
Attitude (2)	4.71	.94	.53**	-				
Subjective norm (3)	3.05	1.20	.26**	.11**	-			
Perceived behavioral control(4)	5.08	.93	.05	.20**	-.03	-		
Commitment (5)	4.90	1.31	-.18**	.004	-.05	.34**	-	
Behavioral disinhibition (6)	2.21	.50	-.07*	-.06*	-.07*	.21**	.14**	-

Note.

** *p* < .01;

* *p* < .05

### Predicting intentions to change lifestyle

Following recommendations by [43] and others [44, 45], we used centered scores for predictors and raw scores for the criterion in a hierarchical multiple regression analysis predicting intentions to lifestyle change (reporting both unstandardized and standardized regression coefficients). More specifically, as can be seen in Table 2, the intention to change to a sustainable, healthy lifestyle was regressed on the constructs from the Theory of Planned Behavior–attitude, subjective norm, and perceived behavioral control in Step 1. Underscoring the predictive validity of the TPB variables and thus replicating past research (e.g., [24]), of these, attitude and subjective norm significantly predicted intentions to change lifestyle. Paralleling the correlational results, the regression coefficient of perceived behavioral control failed to reach significance.

**Table 2:** Hierarchical multiple regression analyses predicting intentions to change lifestyle.

	Step 1			Step 2				Step 3			
	B	β	*R* ^2^	B	β	*R* ^2^	*F*-change (df)	B	β	*R* ^2^	*F*-change (df)
Attitude	.77**	.52**	.320	.75**	.51**	.349	44.77** (1)	.75**	.50**	.350	2.00 (2)
Subjective Norm	.23**	.20**		.23**	.19**			.23**	.20**		
Perceived Behavioral Control	-.07	-.05		.03	.02			.03	.02		
Commitment to Past Behavior				-.20**	-.18**			-.19**	-.18**		
Behavioral Disinhibition								-.01	-.003		
Behavioral Disinhibition x Commitment to Past Behavior								.10*	.05*		

Note:

**p < .001,

* *p <* .05

Commitment to past behavior was added to this set of TPB predictors in Step 2, and contributed significantly and negatively to the intention to change lifestyle over and above the two significant TPB predictors—attitudes and subjective norm. The addition of this single predictor significantly raised *R* ^2^ compared to Step 1 (*F* (1, 995) = 44.77, *p* < .001).

To examine whether the prediction by commitment (accounting for the three TPB control variables) was qualified by individual differences in behavioral disinhibition, we included this construct and the interaction between disinhibition and commitment to past behavior in Step 3 of the hierarchical multiple regression analysis. In this final step, commitment to past behavior still contributed significantly and negatively (95% CI [-.247, -.132]) to the intention to change lifestyle when accounting for the two significant TPB predictors—attitudes (95% CI [.669, .823]) and subjective norm (95% CI [.171, .290]). In line with expectations, results of this step also showed that while disinhibition in itself was inconsequential in predicting intentions to lifestyle change, the interaction reached significance (*p* < .05, 95% CI [.002, .202]). Notably, we also checked whether behavioral *activation* might play a similar role as that postulated for behavioral disinhibition by performing a set of regression analyses now including the interaction terms between each of the three BAS dimensions (BAS drive, fun-seeking and reward responsiveness) and commitment to past behavior. None of these interaction terms reached significance (all *p’* s ≥ .09).

**Fig 1:**
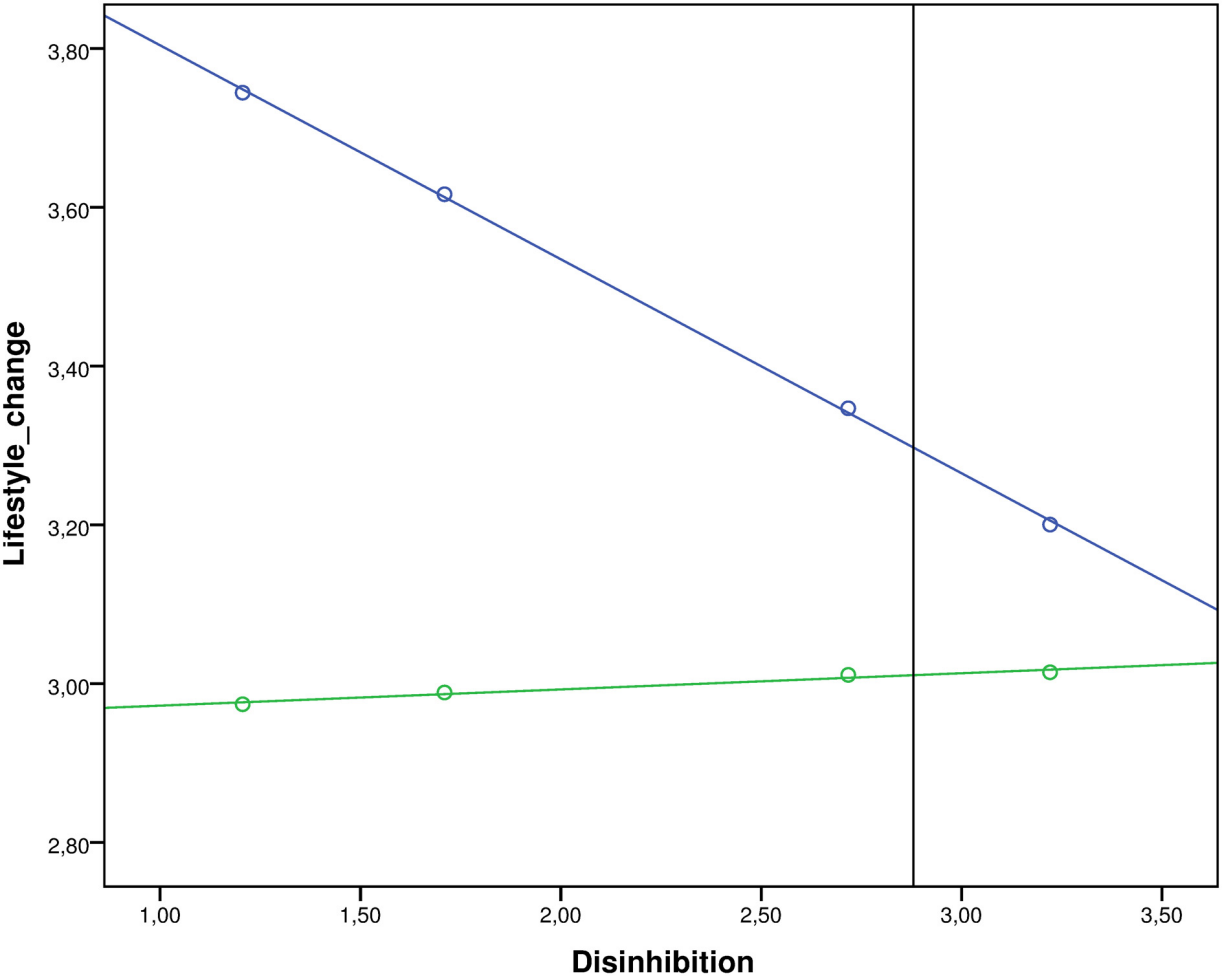
Regression lines of simple slopes of low vs. high commitment at different levels of behavioural disinhibition with Johnson Neyman point. Blue line represents simple slope of low commitment (evaluated at -1 SD from the mean), green line represents simple slope of high commitment (evaluated at +1 SD from the mean). Points represent simple effects of low vs. high levels of commitment at *M*_disinhibition_ -2 SD, -1SD, +1 SD and +2SD. Vertical line indicates Johnson Neyman point (at disinhibition = 2.88). Area to the left of this line indicates region of values of disinhibition for which the simple effect of commitment is significant. Area to the right indicates region of values of disinhibition for which the simple effect of commitment is attenuated and thus not significant.

To further probe the interaction between behavioral disinhibition and commitment to past behavior, we employed the Johnson-Neyman technique [46, 47] to identify the range of values of behavioral disinhibition for which the simple effect of commitment to past behavior was significant, or, conversely, for which this simple effect was attenuated and ceased to contribute negatively to the intention to change lifestyles (using raw values for ease of interpretability). This analysis showed that commitment to past behavior predicted inhibited intentions to change lifestyles only among participants who scored below a value of 2.88 on the behavioral disinhibition scale (B_JN_ = -.11, SE = .06, *p* = .05). For participants with a tendency for behavioral disinhibition higher than this value (the range of values starts at +1.34 *SD* from the mean) this negative contribution was attenuated and commitment to past behavior did not significantly hinder intentions to lifestyle change (see Fig 1).

## Discussion

Using a large (N = 1000) community sample, the present study’s objectives were to examine the role of commitment to past habitual behavior (i.e., to one’s current lifestyle) as a key hindrance to intentions to change lifestyles to a more sustainable, healthy alternative. Moreover, we aimed to assess the extent to which individual differences in the tendency to engage in behavioral disinhibition might overcome these adverse effects.

The results of our study confirmed both notions. More in particular, even when accounting for the contribution of the two significant predictors from the set of established predictors of intentions to change behavior–attitudes, and subjective norms [22, 23]–commitment to past behavior significantly and negatively predicted people’s intentions to change lifestyle. Hence, this motivational “pull of the past” [16] constitutes yet another factor that explains why lifestyle change–a necessity when the aim is to curb the trend toward obesity and unhealthy living [7]–is so problematic for many people.

The reader might wonder why we included perceived behavioral control in all steps of the regression analysis despite its influence failing to reach significance in Step 1. We retained this variable for two substantive reasons: 1. to be consistent across steps in our series of stepwise regression analyses in order for results to remain comparable and 2. to allow for the possibility that any of the predictors added in either Step 2 or Step 3 would have functioned as a *suppressor* thus boosting the contribution of perceived behavioral control (which they did not). Please note that this latter possibility was actually fairly plausible given the correlation between disinhibition (entered at Step 3) and perceived behavioral control. Although the cross-sectional nature of the present data precludes an unequivocal answer, the significance and sign of this correlation tentatively suggest that perceptions of behavioral control may underlie behavioral disinhibition, such that people may engage in disinhibition to the extent that they still experience a sufficient level of behavioral control. Future research might examine this intriguing possibility more in detail.

To our knowledge, the present research is the first to actually zoom-in on the role of commitment to past behavior and to demonstrate its importance for understanding lifestyle change. However, our findings also show that some indeed “have what it takes” to overcome the problem posed by commitment. Hence, this is also the first study to show that that problem is qualified, such that it is mainly an issue for individuals with a tendency for behavioral inhibition. In contrast, for those with a high inclination for behavioral disinhibition, we found the opposite: the burden of past behavior will not spillover to inhibit intentions to future behavior change. Interestingly, this attenuation of the adverse contribution of commitment was observed for the range of values of disinhibition starting at a value over 1 SD above the mean, but not excessively so, thus suggesting that its role is not confined to people with an extreme or outlying inclination for behavioral disinhibition. As such, the present findings are among the very few that point to the “bright side” of behavioral disinhibition in promoting improved self-regulation and prosocial behavior (e.g., [48]), and so complements the vast reservoir of studies that have emphasized the “dark side” of disinhibition, fueled by findings suggesting that the ensuing impulsivity promotes largely unhealthy and risky behaviors, e.g., [34, 35, 36, 37].

While we aimed to highlight the compensatory power of behavioral disinhibition to overcome the paralyzing effects of past behavior, it is also apt to point out that the “flipside” of the effect nicely aligns with past findings. That is, for people lower in behavioral disinhibition, and so higher in BIS activity, the tendency to experience commitment to past behavior indeed lowers the odds of behavior change to a healthier alternative (cf. [29]).

We did not find a contribution similar to that of disinhibition for its pendants on the ‘behavioral activation’ (BAS) side–BAS drive, fun seeking and reward responsiveness, but that does not necessarily mean that these constructs have no role to play when it comes to fostering healthy behavior change. Rather, as argued in the introduction, when the object of change is a comprehensive, general lifestyle, inhibition might be more suitable (as in the present case). However, when change pertains to specific behaviors under the burden of specific past behavior (e.g., changing eating brownies in front of the TV to eating an apple in front of the TV), it might very well be that any or more of the BAS dimensions might outperform behavioral disinhibition. This might be an interesting avenue for future research.

Note that while the effect size of the influence of commitment is substantial, the effect size of the interaction between behavioral disinhibition and commitment to past behavior is modest. Nevertheless, even small effect sizes may signal interesting effects [49] and we feel the present results are a case in point. More specifically, it is interesting to note that the 95% confidence interval for the interaction effect does not include zero, thus suggesting that while small, the effect may still be non-trivial. Next, smaller effect sizes are to be expected when the a priori postulated effects are subtle and interaction effects involving chronic individual difference characteristics (rather than more salient situational manipulations) are typically of such a subtler nature, due to their assessment being driven not only by stable person-specific attributes, but also by situational influences because measurement always takes place in a specific situational context [50]. Indeed, the fact that the present study involved a survey, completed by participants in their home-environment, may have induced higher levels of ‘noise’ in the data, than one would expect in a controlled lab-setting thus possibly lowering effect sizes. Finally, to properly assess the merits of the effect size associated with the interaction-effect, we need to take its nature into account. That is, as reported in the results section, probing the interaction using the Johnson Neyman technique [47] revealed that the simple effect of commitment on intentions to change lifestyle turns from significant to non-significant (i.e., is attenuated) at the value of disinhibition of 2.88, i.e., at 1.34 SD from the mean. This implies that the moderation is driven by a smaller proportion of the sample (i.e., 12.2%) than what would be observed in cases of more symmetric ‘spotlight analyses’ where a simple effect is–say- significant at -1 SD from the mean but non-significant at +1SD from the mean. Still, our findings show that while commitment to past behavior is indeed a formidable obstacle to behavior change, for one out of every 8 participants in our sample it is not, since its influence is attenuated as a function of their higher levels of behavioral disinhibition. Given the challenge of designing successful interventions that target entire health-related lifestyles, this result, while subtle, might still be considered interesting, relevant and worthwhile.

Finally, it should be noted that while we sometimes use terms such as “effect” (as when discussing effect size measures), we should keep in mind that the present results are based on self-reported, cross-sectional data and hence correlational in nature. Hence, to more firmly draw conclusions about the causal nature of the proposed relations, future research might well use a longitudinal design allowing for a more unequivocal assessment of the causal role of the proposed antecedents (i.e., TPB predictors and commitment to past behavior) and moderator (i.e., behavioral disinhibition) in shaping intentions to lifestyle change and ultimately -via these intentions- in affecting actual behavior change.

Although in and of itself disinhibition does not promote intentions to positive lifestyle change, our findings point to an intriguing conclusion, namely that the people that are most vulnerable to engage in impulsive and unhealthy indulgence, i.e., those with a tendency for behavioral disinhibition, are also among the first to overcome the impediment posed by commitment to past behavior and change an unhealthy lifestyle into a more healthy, sustainable one.

## Supporting Information

S1 Data (SAV)

S1 Text Full questionnaire and instructions. (DOC)
