# Supplementary material for: Behavioral Disinhibition Can Foster Intentions to Healthy Lifestyle Change by Overcoming Commitment to Past Behavior
Source: PLoS One. 2015 Nov 11;10(11):e0142489. doi: 10.1371/journal.pone.0142489 (PMC4641622; doi:10.1371/journal.pone.0142489)
Supplement: S1 Text — (DOC) [file pone.0142489.s002.doc]

# Instruction:

# Welcome as a participant in a research project at BI Norwegian Business School. Thank you for taking time to participate in our survey, it will take about 15 minutes to complete. All responses will be handled anonymously, and the results from this research will only be used for scientific purposes and will not be distributed further. There are no right or wrong answers – just your honest opinion.

# The current study is about changing lifestyle to a more sustainable lifestyle.By lifestyle, we mean all the activities you do to promote a healthy living and to promote your individual well-being. This includes a way of life which involves having the right amount of food, drinking, and exercises so that you as a person, are in a state of physical, social and mental well being, whilst having the ability to meet the demands of the environment without undue fatigue.

Questionnaire:

**7-point bipolar scales:**

I would rate myself:

1. Unintelligent – intelligent (e.g., unintelligent 1 2 3 4 5 6 7 intelligent)
2. Imperceptive – perceptive
3. Unanalytical – analytical
4. Unreflective – reflective
5. Uninquisitive – curious
6. Unimaginative – imaginative
7. Uncreative – creative
8. Uncultured – cultured
9. Unrefined – refined
10. Unsophisticated - sophisticated

**7 point Likert statements: 1 – fully disagree – 7 = fully agree**

1. I value what I have higher than what I can have.
2. I value my current lifestyle more than any new lifestyle
3. My current lifestyle means a lot to me.
4. I can estimate the value of what I have better than similar new objects.
5. Not being careful enough has gotten me into trouble at times.
6. It is the fear of loss that keeps me from adopting new services

**7-point scales:**

1. Growing up, would you ever “cross the line” by doing things that your parents would not tolerate?
2. Did you get on your parents nerves often when you were growing up?
3. How often it did you obey rules and regulations that were established by your parents?
4. Growing up, did you ever act in ways that your parents thought were objectionable?

**7 point Likert statements: 1 – fully disagree – 7 = fully agree**

Changing my lifestyle to become more sustainable is something . . .

1. I do frequently.

2. I do automatically.

3. I do without having to consciously remember.

4. that makes me feel weird if I do not do it.

5. I do without thinking.

6. that would require effort not to do it.

7. that belongs to my (daily, weekly, monthly) routine.

8. I start doing before I realize I’m doing it.

9. I would find hard not to do.

10. I have no need to think about doing.

11. that’s typically “me.”

12. I have been doing for a long time.

**7 point LIKERT scale anchored Fully disagree – Fully agree**

1. I will be able to achieve most of the goals that I have set for myself
2. When facing difficult tasks. I am certain that I will accomplish them.
3. In general, I think that I can obtain outcomes that are important to me
4. I believe I can succeed at most any endeavour to which I set my mind
5. I will be able to successfully overcome many challenges
6. I am confident that I can perform effectively on many different tasks
7. Compared to other people, I can do most tasks very well
8. Even when things are tough, I can perform quite well.

**7-point semantic differential items**

1. I think it is bad/good to change lifestyle
2. I think it is foolish/wise to change lifestyle
3. I think it is harmful/beneficial to change lifestyle
4. I think it is unpleasant/pleasant to change lifestyle
5. I am favourable/unfavourable to change lifestyle
6. I think it is punishing/rewarding to change lifestyle

**7 point scales**

1. With respect to adopting a new lifestyle, I would like to do what my closest friend(s) think(s) I ought to do.
   1. Anchored very much – very much not
2. Regarding adopting a new lifestyle, I want to do what my friend(s) think(s) I should do.
   1. Anchored unlikely – likely
3. How much do you want to do what your closets friend(s) think(s) you should do?
   1. Anchored not at all – very strongly

**Anchored Strongly disagree – Strongly agree: 7 point LIKERT scale**

1. I would like to adopt a new lifestyle today, if possible
2. I will try to adopt one new element of a new lifestyle as soon as I can
3. I am likely to be of the first of my friends to adopt a new lifestyle.

**4-point scales:**

**1 = very true for me
  2 = somewhat true for me
  3 = somewhat false for me
  4 = very false for me**

1.  A person's family is the most important thing in life.
2.  Even if something bad is about to happen to me, I rarely experience fear or nervousness.
3.  I go out of my way to get things I want.
4.  When I'm doing well at something I love to keep at it.
5.  I'm always willing to try something new if I think it will be fun.
6.  How I dress is important to me.
7.  When I get something I want, I feel excited and energized.
8.  Criticism or scolding hurts me quite a bit.
9.  When I want something I usually go all-out to get it.
10.  I will often do things for no other reason than that they might be fun.

11.  It's hard for me to find the time to do things such as get a haircut.
12.  If I see a chance to get something I want I move on it right away.
13.  I feel pretty worried or upset when I think or know somebody is angry at me.
14.  When I see an opportunity for something I like I get excited right away.
15.  I often act on the spur of the moment.
16.  If I think something unpleasant is going to happen I usually get pretty "worked up." *
17.  I often wonder why people act the way they do.
18.  When good things happen to me, it affects me strongly.
19.  I feel worried when I think I have done poorly at something important.
20.  I crave excitement and new sensations.

21.  When I go after something I use a "no holds barred" approach.
22.  I have very few fears compared to my friends.
23.  It would excite me to win a contest.
24.  I worry about making mistakes.
